# Effect of Orally Administered Collagen Peptides from Bovine Bone on Skin Aging in Chronologically Aged Mice

**DOI:** 10.3390/nu9111209

**Published:** 2017-11-03

**Authors:** Hongdong Song, Siqi Zhang, Ling Zhang, Bo Li

**Affiliations:** 1 Beijing Advanced Innovation Center for Food Nutrition and Human Health, College of Food Science and Nutritional Engineering, China Agricultural University, Beijing 100083, China; songhd@cau.edu.cn (H.S.); zsq199312@163.com (S.Z.); zhanglingys@outlook.com (L.Z.); 2 Beijing Higher Institution Engineering Research Center of Animal Product, Beijing 100083, China

**Keywords:** collagen peptides, bovine bone, proline, skin aging, chronologically aged mice, antioxidative enzymes

## Abstract

Collagen peptides (CPs) have demonstrated to exert beneficial effects on skin photoaging. However, little has been done to evaluate their effects on chronologically aged skin. Here, the effects of CPs from bovine bone on skin aging were investigated in chronologically aged mice. 13-month-old female Kunming mice were administered with CPs from bovine bone (200, 400 and 800 mg/kg body weight/day) or proline (400 mg/kg body weight/day) for 8 weeks. Mice body weight, spleen index (SI) and thymus index (TI), degree of skin laxity (DSL), skin components, skin histology and antioxidant indicators were analyzed. Ingestion of CPs or proline had no effect on mice skin moisture and hyaluronic acid content, but it significantly improved the skin laxity, repaired collagen fibers, increased collagen content and normalized the ratio of type I to type III collagen in chronologically aged skin. CPs prepared by Alcalase performed better than CPs prepared by collagenase. Furthermore, CPs intake also significantly improved the antioxidative enzyme activities in skin. These results indicate that oral administration of CPs from bovine bone or proline can improve the laxity of chronologically aged skin by changing skin collagen quantitatively and qualitatively, and highlight their potential application as functional foods to combat skin aging in chronologically aged process.

## 1. Introduction

The impact of aging on the appearance and function of skin has received increasing attention in recent decades. It is widely accepted that skin aging is distinguished into chronological skin aging and skin photoaging [1]. Skin photoaging is caused by solar radiation and it is common in sunlight-exposed skin, especially in the face [2]. Therefore, skin photoaging could be prevented or decreased by photo-protection. The common clinical signs of photoaged skin include deep and coarse wrinkles, dryness, sallowness and laxity [2,3]. In contrast, chronological skin aging is caused by passage of time and it takes place all the time in whole-body skin, including facial skin. Chronologically aged skin is characterized by fine wrinkling and laxity [3]. Chronological skin aging accounts for a great part of skin aging and it is more common than skin photoaging in dark skinned individuals and females [2]. A youthful appearance is considered to play an important role in keeping self-esteem and social relations [4]. Therefore, there is increasing demand for anti-aging interventions to delay or even reverse signs of skin aging.

The use of diet supplements to improve the appearance and function of aged skin has received growing attention. Many dietary components, such as polyphenols [5], vitamins [6], fatty acids [7], trace minerals [8] and proteins [9], have reported to exert beneficial effects on aged skin and have been used as nutraceuticals or functional foods in many counties and regions. Recently, researchers have paid much attention to protein hydrolysates as potential dietary supplements. Collagen is the main structural protein of the different connective tissues, such as skin, bone, cartilage and tendons, and has been widely used in the medicine and food industries. Collagen peptides (CPs) are the enzymolysis product of collagen or gelatin and they are used as important active components because of their various bioactivities, high bioavailability and good biocompatibility [10,11,12]. Several studies have demonstrated the beneficial effects of CPs ingestion on skin photoaging. Oral administration of CPs from fish skin had obvious protective effects on photoaging skin, including improving moisture retention ability, repairing the endogenous collagen and elastin protein fibers [13,14,15]. In addition, clinical trials have also demonstrated that the beneficial effects of CPs intake on facial skin, including improving facial skin elasticity, reduce skin dryness and wrinkles, and increase the collagen content of the skin dermis [16,17]. However, little work was performed to evaluate the effects of CPs intake on chronologically aged skin.

Bovine bone is the main by-products in the bovine processing industry and has been widely used as raw material to obtain high-quality gelatin [18]. Although there are some concerns with mad cow disease in Europe and the United States, bovine bone is still one of the most abundant sources of gelatin and accounts for 23.1% of the gelatin production [19]. Therefore, bovine bone is an abundant and high-quality raw material used to prepare CPs. The biological effect of CPs from bovine bone is mainly concentrated on its beneficial effect on bone metabolism, including inhibition of bone loss and improvement of osteoarthritis [20,21]. However, there is limited knowledge about the effect of CPs from bovine bone on skin aging. Therefore, preparing CPs from bovine bone and further evaluating its effect on skin aging is a good way to utilize the by-products for an economical and environmental advantage.

The functional activities of protein-derived hydrolysates or peptides are greatly impacted by their molecular structure and weight, which are highly affected by their processing conditions and especially enzyme specificity [15,22]. Alcalase is a common protease and widely used to prepare protein hydrolysate or peptides. It is a typical endoprotease and preferentially cleaves sites containing hydrophobic residues, such as Ala, Leu, Val and Phe. Bacterial collagenase is a protease that hydrolysates collagen. It has a preference for X-Gly (X is usually a neutral amino acid) bond of the -Gly-Pro-X-Gly-Pro-X- repeating sequence in the collagen molecule [23,24]. Collagenase has a great promise in collagen processing industry. Considering enzyme specificity, the molecular structure or sequences of peptides produced by these two enzymes may be different, which may greatly impact their effects on chronologically aged skin.

The objective of the present study is to investigate the effects of CPs from bovine bone on skin aging based on the chronologically aged model. Bovine bone was employed as a raw material to prepare different CPs using Alcalase and collagenase. Then, the effects of CPs from bovine bone on chronologically aged skin were investigated in chronologically aged mice by analyzing the skin histology, skin components and antioxidative indicators. The results showed that oral administration of CPs from bovine bone has beneficial effects on chronologically aged skin by improving the skin laxity, but it had no on moisture retention of skin. CPs prepared by Alcalase performed better than CPs prepared by collagenase.

## 2. Materials and Methods

### 2.1. Materials and Chemicals

Alcalase was purchased from Novozymes (Beijing, China). Proline (food grade) and bacterial collagenase were purchased from Sigma-Aldrich (St. Louis, MO, USA). The bicinchoninic acid (BCA) protein assay kit was purchased from Beijing Solarbio Science and Technology Co., Ltd. (Beijing, China). Commercial kits used for determining hydroxyproline (Hyp), type I and type III collagen, hyaluronic acid (HA), superoxide dismutase (SOD), catalase (CAT), and malondialdehyde (MDA) were purchased from Jiancheng Inst. of Biotechnology (Nanjing, China). All other chemicals used in the study were of analytical grade or better.

### 2.2. Collagen Peptides (CPs) Preparation

Gelatin was extracted from bovine bone with hot water. Briefly, the bovine bone was treated in boiling water for 6 h, followed by removing bone using gauze filter. The filtrate was cooled, defatted and centrifuged at 4500× *g* for 15 min with a refrigerated centrifuge (TGL-185, Pingfan Co., Ltd., Changsha, China). After centrifugation, the upper soluble fractions were collected and freeze-dried to obtain the gelatin. The gelatin was enzymatically hydrolyzed by the Alcalase at pH 8.0 for 4.0 h to obtain collagen peptides (named ACP), and the collagenase at pH 7.5 for 3.0 h to obtain collagen peptides (named CCP). Finally, the hydrolysates were dialyzed to discard salt and free amino acids, freeze-dried and stored at −80 °C until use.

### 2.3. Molecular Weight Distribution

The molecular weight distribution of CPs was measured using a Shimadzu LC-15C high performance liquid chromatography (HPLC) system (Shimadzu, Tokyo, Japan) equipped with a TSK gel G2000 SWXL column (7.8 × 300 mm, Tosoh, Tokyo, Japan). Samples were loaded onto the column and eluted with 45% (*v*/*v*) acetonitrile containing 0.1% (*v*/*v*) trifluoroacetic acid at a flow rate of 0.5 mL/min and monitored at 214 nm at room temperature. A molecular weight calibration curve (*y* = −0.1881*x* + 6.5867, *y*: log MW, *x*: time, *R*^2^ = 0.9954) was obtained from the average retention times of the following standards: Gly–Ser (146 Da), Asn–Cys–Ser (322 Da), Trp–Pro–Trp–Trp (674 Da), bacitracin (1423 Da) and aprotinin (6512 Da) [15].

### 2.4. Amino Acid Composition

The samples were hydrolyzed in 6.0 M HCl at 110 °C for 24 h. After phenylisothiocyanate (PITC) derivatization reaction, the amino acid composition was analyzed by a Shimadzu LC-15C high performance liquid chromatography (HPLC) system (Shimadzu, Tokyo, Japan) equipped with a reverse Zorbax SB-C18 column (4.6 × 250 mm, Agilent, Santa Clara, CA, USA). The mobile phase consisted of (A) 10 mM phosphate buffer solution (pH 6.9) and (B) 100% acetonitrile and the flow rate was 1.0 mL/min. The gradient was programmed as follows: 0–5 min, 5–10% B; 5–25 min, 10–17% B; 25–45 min, 17–35% B; 45–48 min, 35–100% B; 48–50 min, 100% B; 50–58 min, 100–5% B; and 58–60 min, 5% B. The detection wavelength was set at 254 nm [15].

### 2.5. Animals, Diets, and Treatments

Animal experiments were carried out under the protocols approved by the Committee for Animal Research of Peking University and followed the Guide for the Care and Use of Laboratory Animals (NIH publication No. 86-23, revised 1996). The present experiment was approved by the Animal Experimental Welfare & Ethical Inspection Committee, the Supervision, Inspection and Testing Center of Genetically Modified Organisms, Ministry of Agriculture (Beijing, China), and was performed in the Experimental Animal Center, Supervision and Testing Center for GMOs Food Safety, Ministry of Agriculture (SPF grade, Beijing, China).

Two-month-old (young mice, 28 ± 2 g, specific pathogen free (SPF) grade) and thirteen-month-old (old mice, 45 ± 5 g, SPF grade) female Kunming mice were purchased from Sibeifu (Beijing) Laboratory Animal Science and Technology Co., Ltd. (Beijing, China). The two-month-old mice were set as young controls (*n* = 10) and were given 0.2 mL normal saline. The thirteen-month-old mice were divided, based on body weight, into 6 groups (*n* = 10/group), including the model group and CPs treatment groups. The model group was given 0.2 mL normal saline; whereas the CPs treatment groups were given 0.2 mL ACP at doses of 200 (ACP-200), 400 (ACP-400) and 800 mg/kg body weight (ACP-800), respectively, and 0.2 mL CCP at a dose of 400 mg/kg body weight (ACP-400). In addition to free access to normal AIN-93M purified diet and water, each group was intragastrically administrated with 0.2 mL of normal saline or CPs once a day for eight weeks. After eight weeks, mice were sacrificed and samples were collected for further treatment and analysis.

### 2.6. Measurement of Degree of Skin Laxity (DSL)

During the period of this study, mice backs were epilated with 6% (*w*/*w*) sodium sulfide 2 days before measuring degree of skin laxity each time. Briefly, the dorsal skin, about 1 cm away from the tail root, was gently stretched by left hand with mice hind limbs off the table top slightly. Right hand controls mouse movement by pulling tail. The stretch length was measured immediately when mice were immobile. The DSL was defined as the following equation: DSL (mm) = stretch length of dorsal skin.

### 2.7. Measurement of Spleen Index (SI) and Thymus Index (TI)

The mice were weighed and sacrificed. Spleen and thymus were excised from the mice and weighed immediately. The spleen index (SI) and thymus index (TI) were calculated according to the following equation: SI or TI (mg/g) = (weight of spleen or thymus)/body weight.

### 2.8. Histological Analysis

After eight weeks, mice were sacrificed and dorsal skin samples were dissected out immediately. 4 skin samples (About 1 cm^2^) in each group were fixed in 4% buffered neutral formalin solution for 24 h, and embedded in paraffin. Serial sections (7 μm) were put onto silane-coated slides and stained with haematoxylin–eosin (HE). The stained sections were further analyzed using an optical microscope. 1 representative image of HE-stained dorsal skin section in each group was presented in part of results.

### 2.9. Measurement of Moisture Content

Mice backs were epilated with 6% (*w*/*w*) sodium sulfide 2 days before sacrificing mice. Dorsal skins were collected after mice were sacrifice and skin moisture was measured immediately. The moisture content of skin sample was determined according to GB/T5009.3-2010, a national standard of China for measuring moisture content. This method was employed to measure moisture content of skin in several previous reports [13,14]. Briefly, about 0.1 g of powdered skin sample was put into weighing bottle and dried in an oven at 105 °C for 4 h. The moisture content was calculated according to the following equation:

Moisture content = (*m*_1_ − *m*_2_)/(*m*_1_ − *m*_3_) × 100 (1)

*m*_1_, *m*_2_ and *m*_3_ is the weight of weighing bottle plus skin sample, weighting bottle plus dry finished skin sample and weighing bottle, respectively.

### 2.10. Determination of Hyaluronic Acid (HA) Content

About 0.1 g skin tissue was powdered in a liquid nitrogen bath and homogenized in pre-cooling saline. After centrifugation at 14,000× *g* for 15 min at 4 °C with a refrigerated centrifuge (TGL-185, Pingfan Co., Ltd., Changsha, China), the supernatant was collected to analyze the hyaluronic acid (HA) content using a commercial HA measurement kit (Nanjing Jiancheng Bio Inst., Nanjing, China).

### 2.11. Determination of Collagen Content

A commercial hydroxyproline assay kit (Nanjing Jiancheng Bio Inst., Nanjing, China) was used to analyze the Hyp content. Briefly, about 0.05 g skin tissue was totally hydrolyzed, oxidized and reacted with dimethyl-amino-benzaldehyde. The end product has a maximal absorption at 550 nm. The Hyp content in the skin was finally determined by comparison with the absorbance of the Hyp standard. The collagen content was calculated according to the Hyp content using a conversion factor of 8.00 [25].

### 2.12. Ratio of Type I to Type III Collagen

Commercial type I and type III collagen assay kits (Nanjing Jiancheng Bio Inst., Nanjing, China) were used to analyze the relative content of type I and type III collagen. The ratio of type I to type III collagen was calculated according to the following equation: ratio of type I to type III collagen = content of type I collagen/content of type I collagen.

### 2.13. Antioxidant Indicators Analysis

Skin tissue were powdered in a liquid nitrogen bath and homogenized with 9 weights of pre-cooling saline. Homogenate was centrifuged at 14,000× *g* for 15 min at 4 °C with a refrigerated centrifuge (TGL-185, Pingfan Co., Ltd., Changsha, China) to collect the supernatants. Total protein concentration was determined using a bicinchoninic acid (BCA) assay kit (Solarbio, Beijing, China). The SOD activity, CAT activity and malondialdehyde MDA content (expressed as MDA equivalents) were analyzed using the corresponding enzyme-linked immunosorbent assay (ELISA) kit (Nanjing Jiancheng Bio Inst., Nanjing, China) according to the manufacturer‘s instructions and the results were expressed in U/mg protein or nmol/mg protein.

### 2.14. Statistical Analysis

Results are expressed by the means ± standard deviation (SDs). Comparisons between two groups were analyzed by Student’s *t*-test. Differences between the means of the individual groups were analyzed using the analysis of variance (ANOVA) with Duncan’s multiple range tests. A difference was considered statistically significant when *p* < 0.05. All computations were performed with SPSS Statistics 19 (IBM, Chicago, IL, USA).

## 3. Results

### 3.1. Characterization of Collagen Peptides

**Figure 1:**
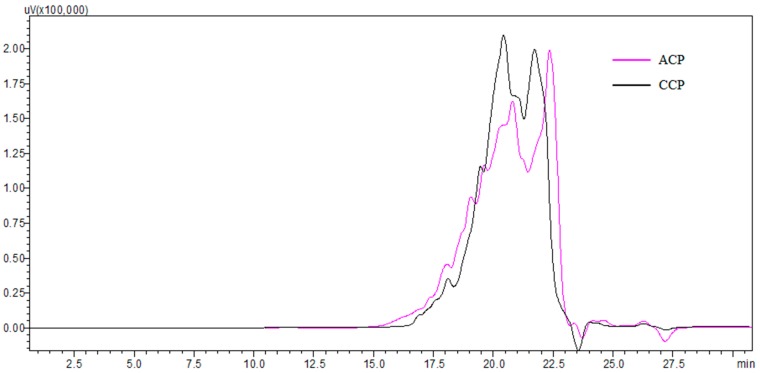
The molecular weight distributions of collagen peptides.

Alcalase and collagenase (two optimized enzymes in our prior study) were used for producing different collagen peptides (named ACP and CCP, respectively). The molecular weight distributions of ACP and CCP are shown in Figure 1. ACP and CCP had a similar molecular weight distribution. Both ACP and CCP mainly consisted of peptides in molecular weight ranges of <500 Da (more than 50%), and the peptides of <1000 Da accounted for approximately 70% and 74%, respectively.

The amino acid compositions of ACP and CCP are shown in Table 1. ACP and CCP had similar amino acid compositions. Gly is the most dominant amino acid in ACP and CCP, which is consistent with the Gly-X-Y repeating sequence in the collagen macromolecule. In addition, ACP and CCP are also rich in Pro, Glu, Phe, Arg and Thr.

### 3.2. Degree of Skin Laxity

As summarized in Table 2, degree of skin laxity (DSL) of young (Y) group was increased during the experiment period but significantly lower than that of model group (old mice), which indicated that skin laxity was increased in an age-dependent manner. During the 8 weeks, the DSL of mice in CPs-treated groups (ACP and CCP groups) decreased over time compared with that in week 0. Significant differences in DSL were seen between ACP-400 group and time-matched model group at week 6 (*p* < 0.05), and the DSL of ACP-400 had no significant difference with that of young group. Furthermore, when the time of oral intake of ACP was as long as 8 weeks, the DSL of all ACP-fed groups decreased to the level of young group (*p* > 0.05), and some of groups (ACP-800 and CCP-400) were even better than the young group. Similarly, oral administration of proline at a dose of 400 mg/kg body weight also reduced the DSL with a significant difference observed compared with the time-matched model group at week 8 (*p* < 0.05).

### 3.3. Body Weight, Spleen Index (SI) and Thymus Index (TI)

The body weight of young group was increased during the experiment period, whereas that of model group remained stable (Table 3). Treatment with ACP (200, 400 and 800 mg/kg body weight), CCP and proline (400 mg/kg body weight) for 8 weeks caused no statistically significant differences in the body weight compared with the untreated model group. Furthermore, the SI and TI of ACP groups, CCP and proline groups also had no significant difference compared to that of the model group. The body weight and organ indices could be measured to preliminarily determine whether a sample or sample dose had obvious toxicological effects on the animal subjects [26]. There was no obvious atrophy, hyperplasia or swelling of spleen and thymus after CPs and proline intake. Based on these results, it was concluded that oral administration of CPs from bovine bone at doses of 200–800 mg/kg body weight, or proline at 400 mg/kg body weight for 8 weeks, had no obvious toxicological effects.

### 3.4. Skin Histology

**Figure 2:**
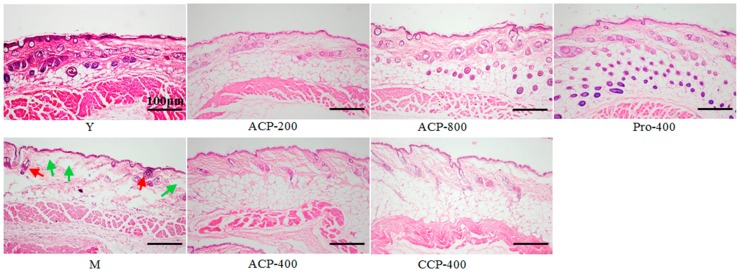
Representative images of haematoxylin–eosin (HE)-stained dorsal skin section from all groups, Y, young group; M, model group (old group); ACP, administrated by collagen peptides which was prepared by Alcalase; CCP, administrated by collagen peptides which was prepared by collagenase; Pro, proline group. 200, 400 and 800 represent administration doses of 200, 400 and 800 mg/kg body weight, respectively. Sebaceous glands and space in dermis tissue are shown as red arrows and green arrows, respectively. Scale bars, 100 μm.

The results of morphological examination of mice dorsal skin are illustrated in Figure 2. Skin collagen fibers in dermis were stained a light red with haematoxylin–eosin (HE). In the model group (old mice), lighter red and more space (green arrow) were observed in the dermis tissue than were those of the young group. There were thinner dermis and less sebaceous gland (red arrow) in the model group compared with the young group. After ACP and CCP intake, the space in the dermis tissue was decreased and the fibers appeared to be denser and more organized compared with the model group. Besides, the number of sebaceous gland was increased when treated by ACP, especially at dose of 800 mg/kg body weight. These results indicated that ACP improved the aged collagen fibers in skin dermis in a dose-dependent manner. Similarly, the sparse, fragmented, and disorganized fibers were also obviously improved and the number of sebaceous gland was greatly increased by the oral administration of proline at a dose of 400 mg/kg body weight for 8 weeks.

### 3.5. Skin Components

**Figure 3:**
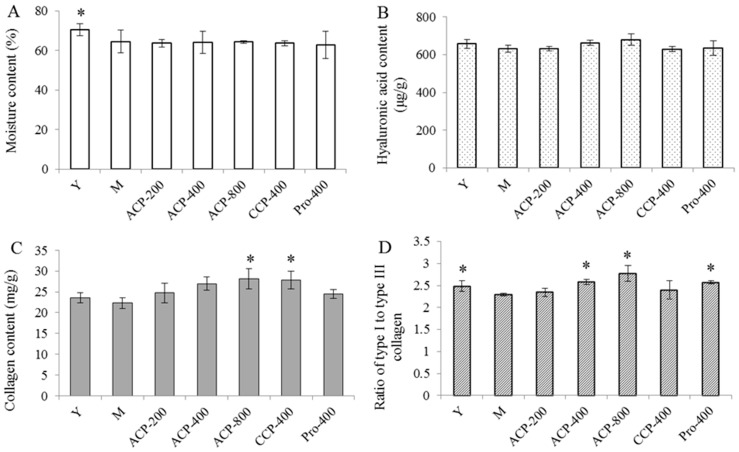
Moisture content (**A**), hyaluronic acid content (**B**), hydroxyproline content (**C**) and ratio of type I to type III collagen (**D**) of chronologically aged mice after the administration of collagen peptides for 8 weeks. Y, young group; M, model group (old group); ACP, administrated by collagen peptides which was prepared by Alcalase; CCP, administrated by collagen peptides which was prepared by collagenase; Pro, proline group. 200, 400 and 800 represent administration doses of 200, 400 and 800 mg/kg body weight, respectively. The values are shown as the means ± SDs (*n* = 10 mice/group). A significant difference was observed at * *p* < 0.05 compared to the time-matched model group.

The results of skin moisture, hyaluronic acid (HA), collagen content and ratio of type I to type III collagen are shown in Figure 3A–D. Skin moisture content and ratio of type I to type III collagen in the model group (old mice) were significantly lower than that in the young group (all *p* < 0.05), indicating that skin moisture content and ratio of type I to type III collagen were decreased with age. Skin HA and collagen contents were also lower than that in the young group, although there was no significant difference observed compared with the model group. Ingestion of CPs (both ACP and CCP) and proline had no significant effect on skin moisture and HA contents compared with the model group. In contrast, oral administration of ACP (200, 400 and 800 mg/kg body weight) caused a dose-dependent increase in the collagen content, and there was a significant difference in the collagen content between the group receiving 800 mg/kg of ACP and the model group (*p* < 0.05). Ingestion of CCP at a dose of 400 mg/kg body weight also increased the collagen content in skin (*p* < 0.05 vs. the model group). However, proline intake at a dose of 400 mg/kg body weight had no significant effect on collagen content compared with the model group. A dose-dependent increase was also observed for ratio of type I to type III collagen in the ACP-fed groups, and there were significant differences between the groups receiving 400 and 800 mg/kg of ACP and the model group (all *p* < 0.05); whereas CCP ingestion at a dose of 400 mg/kg body weight had no significant effect on ratio of type I to type III collagen compared to the model group. Oral administration of proline also significantly increased ratio of type I to type III collagen in skin (*p* < 0.05 vs. the model group).

### 3.6. Antioxidant Indicators

The superoxide dismutase (SOD), catalase (CAT) and malondialdehyde (MDA) content in skin are shown in Table 4. The SOD and CAT activities in the M group were significantly lower in the skin compared to the Y group (*p* < 0.05); whereas the MDA level in the model group was higher than that in the young group (*p* < 0.05). Oral administration of CPs (both ACP and CCP) significantly increased the SOD and CAT activities and reduced the MDA level (all *p* < 0.05 vs. the model group). Besides, the increase of SOD and CAT activities and the decrease of MDA level in ACP-fed groups showed a dose-dependent manner. In contrast, proline ingestion had no obvious significant effect on these three antioxidant indicators.

## 4. Discussion

Skin aging is consisted of chronological aging and photoaging. There are some difference in clinical signs and underlying mechanisms for these two processes [27,28]. Collagen peptides (CPs) have been widely reported to exert beneficial effects on photoaging skin, but few studies was carried out to evaluate their effect on chronologically aged skin. In present study, 13-month-old Kunming mice, equivalent to 45 years old of human life [29], were employed to investigate the effect of CPs on chronologically aged skin. In several previous clinical trials, a daily dose of 2.5 g or 5 g of CPs has been employed in adult subjects, and these doses were considered to be safe [16,17,30]. According to the conversion of animal doses to human equivalent dose (HED) based on the body surface area (BSA) [31], about daily dose of 500 or 1000 mg/kg body weight could be used in mice. In addition, daily doses of 50–200 mg/kg body weight have also been used in several animal experiments [13,14,15]. Therefore, doses of 200, 400 and 800 mg/kg body weight/day were employed in the present study.

Skin laxity is a main feature of natural skin aging and is increased with age [32]. Therefore, skin laxity was dynamically evaluated by measuring the degree of skin laxity (DSL) to observe the effect of CPs intake on chronologically aged skin. An obvious beneficial effect was observed after 8 weeks of CPs intake. Therefore, mice were sacrificed and samples were collected for further treatment and analysis after 8 weeks. Unexpectedly, proline (abundant in collagen) ingestion also significantly improved skin laxity. These results provided guidance for the application of CPs or proline against chronological skin aging. The 8-week duration of CPs ingestion might be equivalent to several years old of human life in terms of life span. But it does not mean that the beneficial effects of CPs could be observed only after several years duration of CPs intake, because several studies have reported that significant beneficial effects of CPs on aging skin could be observed after 6 to 12 weeks in both clinical trials and animal experiments [13,14,15,16,17].

As the main component of the skin dermis, collagen has been reported to be beneficial in improving skin laxity and decreasing the appearance of wrinkles and its reduction in the quantity and quality is a major cause of laxity and wrinkles [33,34]. In the chronologically aged skin, dermal collagen fiber became sparse, fragmented and disorganized [35,36]. However, intake of CPs (both ACP and CCP) repaired collagen fibers and the fibers appeared to be denser and more organized compared to the aged skin. Collagen in skin mainly consists of type I and type III collagen. Collagen production and the ratio of type I to type III collagen is decreased gradually with age [14,28,37,38]. Type I collagen tend to form broader bundles of fibers, while type III collagen forms narrow bundles. A decrease in the diameter and number of the collagen bundles is correlated with the decrease in load and tensile strength reported in aging skin [38]. Oral administration of CPs increased the collagen content and ratio of type I to type III collagen in a dose-dependent manner, which suggested that CPs improved skin laxity by changing skin collagen quantitatively and qualitatively. In contrast, the skin moisture and hyaluronic acid (HA) were not affected by CPs ingestion. HA is a key molecule involved in skin moisture, because it has a unique capacity to bind and retain water molecules [39]. Taken together with the results of the current study, it was concluded that oral administration of CPs had beneficial effect on chronologically aged skin by improving skin laxity, but it had no influence on moisture retention of skin.

Interestedly, ingestion of proline also had beneficial effect on chronologically aged skin in terms of skin laxity, collagen content and ratio of type I to type III collagen. This result of in vivo study was consistent with that of a previous in vitro experiment which demonstrated that proline could increase the collagen synthesis of confluent fibroblasts, but it did not stimulate the proliferation of fibroblasts [40,41]. Watanabe-Kamiyama and coworkers have reported that proline could reach the skin after proline intake [42]. Therefore, we speculated that proline intake exerted beneficial effect on chronologically aged skin by increasing the collagen synthesis of skin fibroblasts.

It has been widely accepted that oxidative stress plays a critical role in initiating and driving the signaling events that result in skin aging. Study has reported that the production of reactive oxygen species (ROS) was increased in photoaged and chronologically aged skin [43]. In addition to directly attacking macromolecules, such as proteins, lipids, DNA and RNA, the excessive ROS also initiate several signaling pathways, including mitogen-activated protein kinases (MAPKs) and nuclear factor kappa-light-chain-enhancer of activated B cells (NF-κB), and further activate transcription factor activator protein-1 (AP-1) [44]. AP-1 induces collagen degradation by upregulating collagen-degraded enzymes such as matrix metalloprotease (MMP)-1, MMP-3 and MMP-9 and downregulating the biosynthesis of collagen. These changes in the skin lead to the phenotype of aged skin [45]. Therefore, antioxidants or free radical scavengers, such as ascorbic acid and polyphenols were reported to improve skin aging by scavenging excessive ROS. Normally, endogenous antioxidant enzymes are able to scavenge the excessive ROS to protect skin tissues from oxidative injuries. SOD and CAT are two antioxidant enzymes that inactivate superoxide anions and hydrogen peroxide, respectively. MDA is a product of lipid peroxidation and is usually quantified to estimate the lipid peroxidation extent induced by ROS. The SOD and CAT activities were decreased and MDA content was increased with age. However, CPs (ACP and CCP) intake could increase SOD and CAT activities and decrease MDA content, indicating that ingestion of collagen peptides from bovine bone had the ability to decrease ROS in skin. The decreased ROS in skin might help to increase the biosynthesis of collagen and decrease the collagen degradation by reducing the MMPs production. Indeed, several previous studies have reported that collagen hydrolysate ingestion could increases skin collagen expression and suppresses MMP-1 and MMP-2 [46,47]. In vitro study had demonstrated that ACP and CCP had high antioxidant capacity base on the hydroxyl radicals and ABTS + scavenging assays (data not shown). In addition, it has been widely reported that nuclear factor E2-related factor 2 (Nrf2)-antioxidant response element (ARE) pathway plays a central role in regulating antioxidant enzymes against oxidative stress [48,49]. Therefore, we speculate that CPs exerted their antioxidant effect in a direct and/or indirect manner. It is also possible that CPs exerted their beneficial effects on chronological aged skin in other ways, as previous studies have reported that Pro–Hyp in human blood after oral ingestion of CPs stimulates fibroblast growth [41,50]. It should be noted that proline intake did not have an obvious effect on skin antioxidant capacity. These results suggested that CPs had more complex action mechanisms underlying anti-aging effect than proline.

Bovine bone is an abundant source of gelatin. The CPs from bovine bone is mainly concentrated on its beneficial effect on bone metabolism. However, the current study found that the CPs from bovine bone also had beneficial effect on skin aging. The CPs prepared in this study mainly consist of oligopeptides (<1000 Da). It was reported that small peptides, especially the di-and tripeptides, are more easily absorbed in the intestinal tract than larger molecules, and oligopeptides are more bioactive than proteins, polypeptides and free amino acids [51,52]. Therefore, we speculated that ACP and CCP are readily absorbed and might exhibit potential biological effects once they are orally administered. Another purpose of this study is to preliminary investigate whether different CPs prepared by Alcalase and collagenase have different effects on chronologically aged skin. Based on the present results, it can be drawn that the beneficial effects of ACP were slightly better than those of CCP. Therefore, Alcalase is a favorable enzyme to produce CPs with beneficial effects on skin aging in food and medical industries. The present result of molecular weight distribution provides a guide for testing and controlling the quality of CPs. Besides, CPs should be protected from oxygen and light because of its easy oxidation.

## 5. Conclusions

In summary, the present study demonstrated oral administration of collagen peptides from bovine bone could improve the laxity of chronologically aged skin by increasing skin collagen content and ratio of type I to type III collagen, but it had no effect on moisture retention of skin. The beneficial effects of collagen peptides prepared by Alcalase (ACP) were slightly better than those of collagen peptides prepared by collagenase (CCP). Another action mechanism underlying the beneficial effects on aged skin of collagen peptides may be involved in increasing the antioxidant properties in the body. Proline intake also improved the laxity of chronologically aged skin but it did not affect the skin antioxidant capacity. These results suggest that collagen peptides from bovine bone and proline are potential dietary supplements for use against skin aging in chronologically aged process.

## Figures and Tables

**Table 1:** Amino acid compositions of collagen peptides.

Amino Acid	Relative Content (g/100 g) ^a,b^	
	ACP	CCP
Asp	5.68	5.17
Glu	10.51	11.53
Ser	3.38	3.17
Gly	19.84	21.28
His	3.27	2.97
Thr	7.90	8.51
Ala	4.09	4.68
Pro	12.47	12.18
Arg	8.61	8.47
Tyr	2.28	1.79
Val	3.06	2.90
Met	1.79	1.27
Cys	2.39	2.08
Ile	4.11	3.93
Leu	0.70	0.08
Phe	9.31	9.36
Lys	0.61	0.62
Total	100.00	100.00

^a^ Expressed as g/100 g total amino acids; ^b^ ACP, collagen peptides prepared by Alcalase; CCP, collagen peptides prepared by collagenase.

**Table 2:** Degree of skin laxity (DSL) of chronologically aged mice after the administration of collagen peptides for 8 weeks.

Group ^a^	Degree of Skin Laxity (DSL, mm)				
	Week 0	Week 2	Week 4	Week 6	Week 8
Y	14.90 ± 2.32 *	19.15 ± 1.57 *	19.75 ± 1.03 *	19.60 ± 0.91 *	20.40 ± 1.48 *
M	22.25 ± 2.40	22.40 ± 1.67	23.80 ± 2.25	22.50 ± 1.30	23.00 ± 1.26
ACP-200	23.05 ± 0.56	23.50 ± 1.64	23.11 ± 1.26	22.00 ± 1.31	21.22 ± 1.47 *
ACP-400	22.45 ± 1.88	21.55 ± 1.78	21.65 ± 1.57	20.15 ± 1.34 *	20.25 ± 1.47 *
ACP-800	22.30 ± 1.81	22.72 ± 2.06	22.05 ± 2.26	21.60 ± 1.78	19.80 ± 0.90 *
CCP-400	22.40 ± 1.57	22.35 ± 1.41	21.25 ± 2.03	22.10 ± 1.92	19.95 ± 1.65 *
Pro-400	21.65 ± 1.23	21.30 ± 2.28	22.80 ± 0.81	21.70 ± 1.00	19.75 ± 1.44 *

^a^ Y, young group; M, model group (old group); ACP, administrated by collagen peptides which was prepared by Alcalase; CCP, administrated by collagen peptides which was prepared by collagenase; Pro, proline group. 200, 400 and 800 represent administration doses of 200, 400 and 800 mg/kg body weight, respectively. The values are shown as the means ± SDs (*n* = 10 mice/group). A significant difference was observed at * *p* < 0.05 compared to the time-matched model group.

**Table 3:** Body weight, spleen index (SI) and thymus index (TI) of chronologically aged mice after the administration of collagen peptides for 8 weeks.

Group ^a^	Body Weight (g)					Spleen Index ^b^ (mg/g)	Thymus Index ^b^ (mg/g)
	Week 0	Week 2	Week 4	Week 6	Week 8		
Y	28.28 ± 0.73	30.27 ± 1.62	31.71 ± 1.30	32.63 ± 1.81	32.70 ± 1.52	3.48 ± 0.69	1.82 ± 0.39
M	47.14 ± 5.39	45.55 ± 4.67	45.55 ± 4.69	46.52 ± 3.40	46.33 ± 3.81	3.83 ± 1.14	1.55 ± 0.53
ACP-200	47.41 ± 5.47	46.28 ± 6.07	46.44 ± 4.41	47.41 ± 4.13	46.83 ± 3.34	3.72 ± 0.06	1.71 ± 0.65
ACP-400	47.50 ± 5.57	46.32 ± 4.91	46.90 ± 4.48	46.64 ± 4.90	46.35 ± 4.90	3.24 ± 1.24	2.06 ± 0.80
ACP-800	47.75 ± 5.67	46.88 ± 4.68	48.36 ± 7.42	49.40 ± 6.63	47.39 ± 6.57	3.86 ± 1.39	1.55 ± 0.62
CCP-400	47.92 ± 5.73	46.82 ± 4.61	48.13 ± 6.55	48.84 ± 4.88	47.38 ± 4.94	3.87 ± 0.94	1.62 ± 0.66
Pro-400	47.24 ± 5.39	46.96 ± 6.14	46.32 ± 5.86	47.65 ± 5.90	47.44 ± 5.69	3.64 ± 1.04	2.02 ± 0.86

^a^ Y, young group; M, model group (old group); ACP, administrated by collagen peptides which was prepared by Alcalase; CCP, administrated by collagen peptides which was prepared by collagenase; Pro, proline group. 200, 400 and 800 represent administration doses of 200, 400 and 800 mg/kg body weight, respectively. The values are shown as the means ± SDs (*n* = 10 mice/group). No significant difference between each administration group and time-matched model group was observed (*p* > 0.05). ^b^ Indicates values at week 8.

**Table 4:** SOD and CAT activities and MDA content in dorsal skin of chronologically aged mice after administration of collagen peptides for 8 weeks.

Group ^a^	SOD (U/mg Protein)	CAT (U/mg Protein)	MDA Equivalents (nmol/mg Protein)
Y	36.594 ± 1.142 *	10.412 ± 1.143 *	2.209 ± 0.278 *
M	26.877 ± 3.880	4.650 ± 1.582	3.135 ± 0.302
ACP-200	38.746 ± 0.753 *	8.324 ± 0.890 *	2.347 ± 0.209 *
ACP-400	39.823 ± 3.410 *	11.327 ± 1.096 *	2.261 ± 0.107 *
ACP-800	40.036 ± 4.820 *	12.012 ± 0.752 *	2.154 ± 0.325 *
CCP-400	39.796 ± 1.211 *	9.354 ± 1.856 *	2.204 ± 0.201 *
Pro-400	32.646 ± 1.691	3.318 ± 0.665	2.456 ± 0.316

^a^ Y, young group; M, model group (old group); ACP, administrated by collagen peptides which was prepared by Alcalase; CCP, administrated by collagen peptides which was prepared by collagenase.; Pro, proline group. 200, 400 and 800 represent administration doses of 200, 400 and 800 mg/kg body weight, respectively. The values are shown as the means ± SDs (*n* = 10 mice/group). A significant difference was observed at * *p* < 0.05 compared to the time-matched model group.
